# Remote treatment of developmental dyslexia: how ADHD comorbidity, clinical history and treatment repetition may affect its efficacy

**DOI:** 10.3389/fpubh.2023.1135465

**Published:** 2024-01-10

**Authors:** Maria Luisa Lorusso, Francesca Borasio, Paola Mistò, Antonio Salandi, Simona Travellini, Mariangela Lotito, Massimo Molteni

**Affiliations:** ^1^Scientific Institute, IRCCS E. Medea, Bosisio Parini, Italy; ^2^Department of Psychology, Catholic University of the Sacred Heart, Milan, Italy; ^3^DISTUM, Department of Humanities, University of Urbino Carlo Bo, Urbino, Italy; ^4^Center of Clinical Developmental Neuropsychology, ASUR, Pesaro, Italy

**Keywords:** dyslexia, remote treatment, ADHD, repeated treatment, multi-component treatment

## Abstract

**Introduction:**

Tachidino is a web-based platform for remote treatment of reading and spelling disorders. The purpose of the present study was to investigate the possible impact of different clinical conditions on the efficacy of treatment. The focus was on possible ADHD comorbidity-related effects on the outcomes of the Tachidino treatment, and the impact of previous treatments, such as speech and language therapy or the repetition of the same Tachidino program.

**Methods:**

136 children with developmental dyslexia received four-weeks treatment via the Tachidino platform. Improvements in reading and writing scores were compared between different subgroups.

**Results:**

No gross differences emerged in treatment effectiveness between groups of children. Children receiving treatment improved significantly more than untreated children.

**Discussion:**

Treatment with Tachidino brought significant benefits for all children, irrespective of comorbidity, clinical history or treatment repetition. Comparison with an untreated control group (waiting list) made it possible to exclude that improvement was due to test–retest learning effects.

## 1 Introduction

In recent years, and mostly during the last months of the COVID-19 pandemic, telemedicine and remote treatments for Developmental Dyslexia have made it possible to keep rehabilitation programs active (1, 2). Developmental Dyslexia (DD) is a heterogeneous learning disorder characterized by a severe and persistent impairment in the acquisition of reading and spelling skills that cannot be due to low intelligence, sensory or neurological damage, or poor educational opportunities (3, 4). DD is currently best explained by multiple-deficits models (5–7) where phonological impairment may be symptomatic of a more basic deficit (8). It is therefore recommended that multiple-deficit models of dyslexia are taken into account in the remediation program choice (9).

The use of remote treatments allows for the maximization of effectiveness in improving reading and writing skills by optimizing the duration and flexibility of the intervention; it also appears feasible and engaging (1, 2, 10).

Tachidino is a web-platform for remote treatment of reading and writing disorders that was developed at Scientific Institute “*E. Medea*” well before COVID-19 outbreak, and has been used as the first-choice, default treatment for patients diagnosed with DD since 2016. Data collected between 2018 and 2022 will be retrospectively analyzed for the purposes of the present study. The treatment consists of a fully automated and individually-tailored training program (2) hosted on a web-based platform that can be accessed by the therapist and by the patient separately. The therapist interface allows the clinician to set all parameters for the treatment (type of stimulus lists, type of exercise, visual parameters and auditory parameters of the stimuli that the child has to process, speed and position of stimulus presentation), to monitor the child’s progress either in real-time or in asynchronous modality and, if necessary, change/adjust the child’s program accordingly. The child can access the program with their own credentials and work following the program set by the therapist in any moment of the day and any day of the week, based on the therapist’s directions but also flexibly adjusting their work to family commitments and needs. The program records speed and accuracy of every single input given by the child and provides real-time graphical representations of the child’s performance both item-wise and list-wise. The child’s responses are automatically collected and processed without the need for an adult to help or monitor the child, nor to give any information about the child’s response accuracy (although it is recommended that adult caregivers monitor the child’s work in terms of time and effort devoted to the activities, to ensure sufficient intensity of treatment on a daily and weekly basis). Tachidino complies with the guidelines for neuropsychological rehabilitation (11) as it is an intensive training requiring daily exercises.

The treatment program is based on a multi-componential and multi-factorial model of dyslexia [see (5, 6)]. More specifically, the treatment combines two types of training, specifically Visual-Attentional Training (12, 13) and Visual Hemisphere-Specific Stimulation (VHSS) (14, 15). In the training, the child is required to identify and select a moving object among other similar moving objects (visual-attentional component), following the same principles of Action Video-Games (AVG), i.e., focusing on a small-size, rapidly and unpredictably moving target, and then to decode or encode words or short text sequences tachistoscopically presented in a specific position of the visual field (VHSS component). Furthermore, the types of stimuli and the types of exercises (which mainly require writing/re-ordering of shortly presented visual stimuli or auditorily presented verbal stimuli) capitalize on either visual analysis or linguistic analysis and call into play more right-hemisphere (RH) encoding and decoding processes focused on the visual characteristics of the verbal stimuli or left-hemisphere (LH) strategies focused on lexical, semantic or morpho-syntactic characteristics of the words (or - in the most complex lists - short combinations of words with different syntactic functions, e.g., article + noun, noun + adjective, verb + noun).

The effectiveness of the program has been shown in comparison with the effects of the combination of the two components of the program (VHSS and AVG) delivered as an in-presence treatment for the same duration and amount of time (16). A recent study focusing on possible differences in Tachidino treatment outcomes specifically related to the age of the child or the severity of the impairments showed significant gains in reading and writing skills after 1 month of treatment in all groups (2). More precisely, however, it was shown that younger children improved more than older children in writing accuracy, and children with more severe initial impairments improved more than children with less severe impairments in reading speed and accuracy and in writing accuracy (2).

Overall, previous investigations of the effectiveness of intervention via the Tachidino platform were based either on comparisons with treatments involving the same theoretical and methodological principles but on an inpatient, in-person modality (16) or on comparisons with other types of treatment (usually also delivered in person) (17), or still on comparisons between subgroups of children differing on age, subtype or severity (2). However, the direct comparison with a control group not receiving treatment was still lacking. In the present study, data from an untreated group of children with DD (waiting list) assessed with the same reading tests at the same time distance as the pre-and post-treatment sessions for the Tachidino group were made available by a collaborating center (ASUR Marche and Urbino University) in a different region.

The first experimental question addressed by the present study was whether the effects obtained with the Tachidino treatment in the group of children with (non-comorbid) DD and not previously treated would be significantly larger than the effects observed in a non-treated group of dyslexic children. This would allow us to exclude that the effects may be due to the mere repetition of the tests after a rather short (one-month) time.

The study further aimed to shed light on the generalizability of the effects of treatment with the Tachidino platform focusing on specific conditions that might reduce its effectiveness, namely the presence of additional disorders (in particular, attention deficit with hyperactivity disorder - henceforth, ADHD) and the delivery of treatment to patients who have already been treated with either the same or different intervention programs.

Indeed, DD has been reported to be frequently comorbid with ADHD (18, 19), with 25 to 40% of children with ADHD presenting with a co-occurring diagnosis of reading disability (20). ADHD is defined as a persistent and pervasive pattern of disruptive behavior characterized by inattention, hyperactivity, and impulsivity (21). Within single-deficit models of DD, the reading disorder may be seen as the primary disorder and the symptoms of ADHD as secondary symptoms caused by reading difficulties, or ADHD may be seen as the primary disorder also causing reading difficulties. Alternatively, the combination of reading disorders and ADHD may be considered as a third disorder etiologically distinct from the two “pure” disorders. However, empirical studies have not provided evidence in favor of any of these hypotheses (22, 23). On the other hand, multiple-deficit models suggest that neurodevelopmental disorders are the result of complex interactions between biological and/or environmental risk and protective factors (5, 24). Comorbidities would thus be explained by shared risk factors that manifest themselves with multiple symptoms. Indeed, the two disorders share common features, such as deficits in attention and response inhibition, processing speed, and working memory (25, 26). However, their underlying pathophysiologies seem to be independent and not causally related (27). It is therefore crucial to understand whether reading interventions that are effective in improving performance in pure disorders are also effective in the case of comorbid disorders (25, 28). Denton et al. (29) compared the effects that an intervention addressing (a) reading difficulties, (b) attentional problems, or (c) combining the two had on the reading skills of children with DD and ADHD. The results showed that (a) and (b) had largely independent effects on attention and reading fluency, while (c) was not better than either treatment alone.

A recent meta-analysis (20) found that rigorous decoding interventions can be considered the eligible treatment in children with ADHD and for at-risk readers with ADHD. Moreover, intensive reading treatments based on phonemic decoding are likely to produce greater reading improvements in children with ADHD (20).

The second experimental question was thus whether the effectiveness of the Tachidino treatment, addressing both decoding and visual-attentional deficits, and having shown its effectiveness with children diagnosed with DD, is also effective when comorbid ADHD is present. Based on previous evidence, it was expected that the effectiveness would be comparable in the comorbid condition. Nonetheless, firstly the specific nature of the attentional component of the treatment, addressing visual–spatial attention rather than the usually addressed executive functions, and secondly, the remote mode of delivery of the treatment, requiring the child’s capacity to organize and control their own activity in a rather independent way, constitute challenges for children with ADHD that make such expectations not obvious at all.

The last experimental question was whether it is possible to obtain continuous gains in reading and writing after multiple cycles of treatment. Recent studies showed that improvements in reading and writing following computerized treatment of DD are still present 6 months after the discontinuation of treatment (2, 30). In addition to these results, however, it would be interesting to explore what further speed and accuracy gains can be obtained by replicating the intervention. Indeed, treatment repetition is suggested and offered in the clinical setting when the two following conditions are present: (i) the child’s reading and writing skills are still below the cut-off of clinical interest (generally, 2 standard deviations below age mean) and (ii) the child’ response to treatment in the first cycle was satisfactory, i.e., clinically significant improvement was observed following the first treatment. In general, a second cycle is offered after a pause of 6 months to 2 years, depending on each single situation (taking into account the child’s motivation and their family’s organization, as well as relevant school-related variables such as changes in school requests and in difficulty levels of the texts to be studied etc.). Since most children reach a reading and writing level within the (low-) normal range after a single treatment cycle with the Tachidino program (2), treatment repetition is proposed to about 20% of the children, who either started from a more severe initial level of impairment or whose first treatment focused on a limited range of goals (e.g., only on reading and not on writing skills), leaving further goals to be addressed subsequently.

Usually, research has reported the outcomes after a single intervention, and only very few studies have explored the impact of treatment repetition. For instance, by repeating the interventions two or three times after a period of no treatment, it seems possible to continue improving reading fluency (31). Another study on Italian children with DD (32) delivered two subsequent cycles of treatment, the first one more focused on visual analysis and the second one more focused on syllable decoding in a sublexical reading approach. The authors reported improvements in the second cycle of treatment for accuracy (of text only, whereas word and nonword reading accuracy did not show any significant improvements in either the first or the second cycle of treatment) that were not present in the first cycle. As to reading speed, improvements were significant in both cycles, but more evident in the first compared to the second one. Based on such (albeit scarce) evidence, we expected that improvement in the second cycle would be still significant, but more limited with respect to that observed in the first cycle.

A different but related instance of treatment replication is represented by children who were treated with different intervention approaches before being offered treatment with Tachidino. Clearly, such children may be different in origin compared to the children who are immediately enrolled in remote treatment programs. In clinical experience, a preliminary treatment with speech and language therapy (SLT) may be offered to younger children or to children whose language pre-requisites (especially phonological awareness) may be particularly weak, so that remote treatment may seem to be too challenging or fatiguing. In fact, most (although not all) children with DD show significant impairments in phonological skills (9, 18, 33–35), and phonological deficits are known to explain a large proportion of variance in literacy skills (from 40% for orthographic skills to 49% for reading speed and 59% for reading accuracy) (36). As the Tachidino treatment program requires decoding of words (or in some cases, single syllables) presented for less than 300 ms, a certain degree of automatization of grapheme-to-phoneme conversion is required to be able to benefit from the program (and to avoid too stressful processing efforts and a too frustrating experience to the child). Moreover, the program requires the visual control of movement and crowding effects, and a general increase in speed of processing and inter-hemispheric integration (15, 37–39). Such processes were shown to be involved in both the aetiology (14, 40) and the remediation (2, 15, 17, 39, 41) of DD, besides phonological awareness. Tachidino could be, therefore, too fatiguing for young children or children with severe phonological or visual attention impairment. For this reason, children who are judged too impaired to access the program immediately may be offered a preliminary training in an SLT setting to first improve their phonological decoding abilities.

Summarizing, the aim of the present study was to compare the gains in reading and in writing of different groups of children with a diagnosis of developmental dyslexia. To assess possible ADHD comorbidity-related differences on the outcomes of the treatment, performances of children with and without ADHD were compared. Moreover, to assess the impact of a clinical history of SLT, the reading and writing gains were compared in children who had received speech and language therapy before treatment with Tachidino and in children who had not received any interventions before. Lastly, the gains obtained with the remote intervention were compared in children who received one or 2 cycles of the program. Based on the studies described above, we expected that all children, irrespective of comorbidity, clinical history or treatment repetition, would significantly benefit from the Tachidino treatment. Nonetheless, we expected the effectiveness of treatment to be reduced in the second versus the first cycle of intervention, as well as after previous treatment with SLT.

## 2 Materials and methods

### 2.1 Participants

Data from a total of 136 children (77 male) aged between 7 and 14 years (mean age = 9.38 years, SD = 1.31) were analyzed for the present study. Children were selected among patients referred to the IRCCS “*E. Medea*” between January 2018 and September 2022. Participants had to fulfil the following inclusion criteria: (a) having been diagnosed with Specific Reading Disorder (ICD-10 codes: F81.0 or F81.3) on the basis of standard inclusion and exclusion criteria (4) (at least one *Z*-score concerning reading/writing speed and/or accuracy below −2, IQ = > 75); (b) absence of comorbidity with other psychopathological conditions (whereas comorbidity with other learning disorders and/or ADHD was allowed). All children were either native Italian speakers or their mastery of Italian was indistinguishable from native.

To the aim of the present study, data from different subgroups of children were analyzed and compared. Depending on the individual profile, the same child could be included in different groups for different comparisons (e.g., one child may have been included in the ADHD group and in the group that repeated the treatment). In particular:

- 82 children (42 male), aged between 7 and 14 years (mean age = 9.56 years, SD = 1.43), belonged to the group of children with DD who received remote treatment via the Tachidino platform but had no comorbid ADHD nor had they been treated before;
- 30 children (17 male) belonging to the previous group, aged between 7 and 14 years (mean age = 9.03 years, SD = 1.33), received 2 cycles of Tachidino treatment;
- 17 children (15 male), aged between 8 and 11 years (mean age = 9.35 years, SD = 1.12), belonged to the group with ADHD in comorbidity;
- 22 children (11 male), aged between 8 and 10 years (mean age = 9.23 years, SD = 0.75), received SLT before starting Tachidino treatment.

An additional group of 16 children (7 male) were recruited at the Center of Clinical Developmental Neuropsychology (ASUR Marche) in Pesaro, Italy, between January 2020 and November 2022. These children were selected following the same inclusion and exclusion criteria, but none of them had ADHD. Moreover, none of these children had received any other treatment for DD before. They were in the waiting list for treatment with Tachidino and were tested at diagnosis and at the beginning of treatment after a period of approximately 3–4 weeks in which they had not received any treatment. Mean age was 10.81 years, SD = 1.83, range 8–14. Due to different research protocols in the two centers, this group was assessed with text, word and nonword reading tests, but they did not undergo any writing tests.

Written parental informed consent was obtained for all children before pre-test assessment. The study was approved by the Local Ethics Committees in accordance with the Declaration of Helsinki.

The data collection of the Tachidino treatment is registered in ClinicalTrials.gov (Code NCT04382482) as an observational study, while the comparison with children recruited at ASUR Marche is registered as NCT04384718.

### 2.2 Neuropsychological assessment

All participants were tested before and after each treatment. Specifically, the same assessment was adopted before and after each cycle of Tachidino. The following tests were administered. All scores are expressed as *z*-scores according to age norms.

1. Text reading: “Prove di rapidità e correttezza nella lettura del gruppo MT” (“Test of speed and accuracy in reading, developed by the MT group”) (42). This test assesses reading abilities for meaningful texts. It provides separate scores for speed and accuracy. Texts increase in complexity with grade level, and norms are provided for each text.
2. Single word and non-word reading: “DDE-2: Batteria per la Valutazione della Dislessia e Disortografia Evolutiva-2” (Assessment battery for Developmental Reading and Spelling Disorders-2) (43). The battery assesses speed and accuracy (number of errors) in reading word lists (96 words) and non-word lists (48 non-words).
3. Single word writing: a writing-to-dictation task was taken from the DDE-2 battery (43), giving accuracy scores according to age norms in writing 48 words. Due to changes in clinical protocols during the collection of data, not all children underwent this writing test.

### 2.3 Procedure

All children were tested individually by a professional psychologist before and after Tachidino treatment. The same assessment procedure was repeated also before and after the SLT and before and after the second cycle of Tachidino. Children with DD who did not carry out any treatment completed the two assessments about 1 month apart. For both treated and untreated groups, there may have been sporadic deviances from the standard interval between pre-test and post-test (planned to be between 3 and 4 weeks), due to holidays, illness or other unforeseen inconveniences.

All participants, with the exception of the control group, took part in the remote intervention program called Tachidino for an average total time of 14 h (range 12–18) over a maximum of 4 weeks. The training program is composed of 20–24 lists of words per week for 3/4 weeks (average number of total lists = 73). The exact duration depends on the child’s level of reading and the intervention does not have a fixed working schedule, so as to adapt to the child’s rhythms and attention capacity, but the children are encouraged to work at least 4–5 days per week in sessions of 20–30 min. The program includes: (a) one pre-treatment, face-to-face meeting in order to define dyslexia subtype, to demonstrate Tachidino use and schedule the first activities, and (b) one intermediate phone call to monitor and motivate correct use of the program. The therapist (a trained psychologist) monitors the child’s progress and adjusts the training program several times per week, and may contact the family in case of evident deviance from the working schedule that had been agreed upon. The psychologist in charge of the assessment was different from the psychologists in charge of monitoring the intervention.

### 2.4 Treatment

The Tachidino treatment aims at improving reading through improvement of both decoding strategies and visuo-spatial attentional abilities. It is a web-based platform including training software, along with systems managing clients’ and professionals’ data and interfaces. Tachidino treatment incorporates two multi-componential principles, specifically Visual-Attentional Training (12, 13) and Visual Hemisphere-Specific Stimulation (VHSS) according to Bakker’s Balance Model (14, 44). Visual-Attentional training, in particular, is inspired by Action Video Games (AVG), which are characterized by an emphasis on peripheral processing and global perception of stimuli moving at high speed and that are spatio-temporally unpredictable, while VHSS aims to stimulate selectively one hemisphere to improve reading.

In the Tachidino program, the child is required to recognize the target candy (a spiral candy) among various candies (distractors) and press the spacebar at the exact moment the target candy is crossing a circle target (fixation point). The word to be decoded/encoded is presented, visually or auditory, only if the child clicks at the right moment, thus ensuring that fixation was in correspondence with the fixation point in the center of the visual field. If the bar is pressed in the target timeframe and in correspondence of the target candy, the word to be decoded/encoded is immediately presented and the child is asked to either write the word on the keyboard or re-order a sequence containing all the correct graphemes in random order.

All visual stimuli are presented at tachistoscopic speed to a visual hemifield in order to stimulate the contralateral hemisphere to a greater degree, or they may also be flashed in the center of the computer screen, involving both hemispheres simultaneously.

The visual hemisphere-specific stimulation is based on a revisit of Bakker’s ‘Balance model’ (14, 44). Each child was classified as a P-, L-, or M-type dyslexic reader based on the persistent over-reliance on specific reading strategies, on reading speed, and on the pattern of reading errors, distinguishing between substantive errors, altering the structure and meaning of the word, and time-consuming errors, allowing for final correct decoding of the word to be read (15, 39). More precisely, each child could be included in one of the three following subtypes:

- P-type (decoding strategies based on accurate perceptual analysis mainly supported by the right hemisphere-RH, resulting in slow but relatively accurate reading);
- L-type (anticipation strategies based on linguistic abilities and mainly supported by the left hemisphere-LH, resulting in relatively fast but inaccurate reading);
- M-type (who strives to use both kinds of strategies but does so inefficiently, resulting in both slow and inaccurate reading) in all other cases (when both error types are present in similar proportion and/or when child is both slow and inaccurate in reading). Classification followed a precise procedure as described in Lorusso and colleagues’ works (2, 15, 39).

The tachistoscopic presentation of visual stimuli depended on the previous classification, and selectively stimulated either RH-specific perceptual analysis using visually complex materials and/or error detection and correction tasks, or LH-specific linguistic anticipation using linguistically inter-related materials and/or anticipation/completion tasks. *M*-type dyslexic readers received the stimulation of the RH first and of the LH at a later stage of treatment, following the stages of natural reading acquisition according to the Balance Model (44). Central stimulation (and/or auditory presentation) was chosen when the target was to improve writing abilities.

Auditory stimuli were presented through Google text-to-speech synthesis, at the desired speed and pitch according to the child’s needs. During auditory presentation of the words, the child is encouraged to extract phonological information from the input, operate phoneme-to-grapheme conversion and match the auditory string with the written visual string. The visual string could be either written by the child or reconstructed based on given sequences of the correct graphemes randomly ordered. In auditory stimulation, the choice of materials and tasks depends on the hemisphere-specific strategy to stimulate. For example, low frequency, concrete, highly imageable words are ideal for RH stimulation, while high-frequency, semantically interconnected, abstract words for the LH one. Auditory presentation is to be considered as a secondary aspect in the training, but relevant for children whose main impairments are in spelling/writing more than in reading skills.

The therapist programs and monitors remotely the child’s activities, either in real time (synchronous mode) or at a different moment (asynchronous mode), and defines all the parameters, such as the graphic background, the laterality of the stimuli, the type of exercise (read/write, read/correct, listen/write, listen/correct), the lists of stimuli, exposure times, the characteristics of the font (type, size, spacing, color) and of the speech synthesis (speed and pitch) (2).

The large number of lists (over 370) and the potentially unlimited combinations of parameters that can be applied to each of them allows for a high degree of personalization of the treatment (exercises and lists as well as the specific parameters are chosen based on the child’s age, subtype, and specific profile of strengths and difficulties which imply different clinical goals). Moreover, they allow for the repetition of the treatment cycle, with new goals and settings adjusted to the updated profile of neuropsychological and reading/writing abilities of the child.

### 2.5 Data analysis

Data were analyzed with SPSS and Jamovi software, according to the following steps. In order to reduce the number of analyses and to obtain more reliable scores, two global *Z*-scores were computed: (i) Global Reading Speed score (the average of speed in text, word and nonword reading), (ii) Global Reading Accuracy score (the average of accuracy in text, word and nonword reading).

In order to compare the effectiveness of treatment in different groups of children, data analysis was carried out with: (a) children with and without ADHD comorbidity, (b) children with and without a clinical history of SLT, and (c) children who underwent the Tachidino treatment twice, for both the first and the second cycle of treatment. Depending on the specific individual profile, the same child could be included in different groups for different comparisons (e.g., one child may have been included in the ADHD group and in the group that repeated twice the treatment). Sporadic data are missing due to technical problems during data collection and recording.

No Bonferroni correction was applied because all analyses were pre-determined and hypothesis-driven, moreover the scores were significantly inter-correlated (mean correlations in difference-scores in the whole group ranged between *r* = 0.311, *p* < 0.001, and *r* = 0.182, *p* = 0.040), and finally some of the children were included in more than one analysis, depending on their specific conditions.

As the study is a retrospective one, sensitivity analyses instead of *a-priori* power analyses (with G-Power) were performed, showing that a sample of 136 participants allows detection of an effect size of 0.12 in a two group, repeated measures ANOVA, within-between interaction, with a power of 0.80. Considering two groups of different sizes, independent sample t-tests (one-tail) sensitivity analyses (with a power of 0.80) showed an effect size of 0.68 for Tachidino vs control group, of 0.65 for ADHD vs non-ADHD group, of 0.58 for SLT vs non-SLT, and of 0.36 for the comparison with the treatment repetition group.

#### 2.5.1 Comparison between control group and Tachidino group

First of all, from the whole sample of children with DD who underwent the Tachidino treatment, we selected a subgroup of children not presenting any comorbidity with other psychopathological conditions or ADHD, and not having been involved in other clinical intervention programs for learning disorders in the past. A series of independent sample t-tests were adopted to compare age and reading and writing skills at the baseline between the control group of children not involved in treatment and this subgroup of children with DD. Subsequently, repeated-measures ANOVAs with group as a between-subject variable and age as covariate were performed on pre-and post-treatment Global scores. When the interaction of Treatment and Group was significant, post-hoc tests were computed first on the two groups separately, comparing pre-and post-treatment scores, and then on the single components of the Global scores (text, word and nonword reading) for the specific parameter (speed or accuracy) showing the significant interaction.

#### 2.5.2 ADHD comorbidity

First of all, independent samples t-tests were adopted to assess possible age and performance differences between the two groups of children. In order to analyze Tachidino training-related changes and the possible treatment-by-group interactions, repeated measures ANOVAs, with presence or absence of ADHD as between-subjects factor, and treatment (pre-treatment/post-treatment scores) as within-subjects factor, were computed. Based on our hypothesis of no differences between the two groups in reading and writing improvements, difference scores (between the post-treatment and the pre-treatment) were further analyzed through TOST (two one-sided *t*-tests) procedures for equivalence testing, comparing the two groups of children (45, 46). Standardized Cohen’s ds of 0.5 were set as upper and lower boundaries.

#### 2.5.3 Previous treatment with SLT

A series of independent samples t-tests were used to evaluate differences of age, reading and writing pre-Tachidino performances between the two groups. Subsequently, repeated measures ANOVAs were used to compare Tachidino training-related changes in children with or without a clinical history of SLT, with presence or absence of previous SLT as between-subjects factor, and treatment (pre-treatment/post-treatment scores) as within-subjects factor. Difference scores (between the post-treatment and the pre-treatment) were calculated and compared in the two groups of children. Considering only the group of children with a clinical history of SLT, a series of paired sample t-tests were used to assess differences between pre-and post-Tachidino in the two groups.

#### 2.5.4 Tachidino treatment repetition

First of all, the pre-test scores of the group of children who repeated treatment was compared with initial scores in the remaining children (excluding children who had previously received SLT), in order to identify possible differences in pre-treatment profiles. As a second step, the pre-treatment scores of the same groups of children were compared to post-treatment scores for the first cycle, so as to highlight significant changes induced by the treatment. Moreover, difference-scores (post-test minus pre-test scores) were computed and compared between the two groups. Finally, in order to analyze treatment-related changes in the 2 cycles, a series of paired-samples t-tests were computed considering only the group of children who underwent the Tachidino treatment twice. Specifically, pre-versus post-treatment difference scores for the first cycle of treatment were compared to pre-versus post difference scores for the second cycle.

## 3 Results

### 3.1 Comparison with control group (untreated)

**Figure 1:**
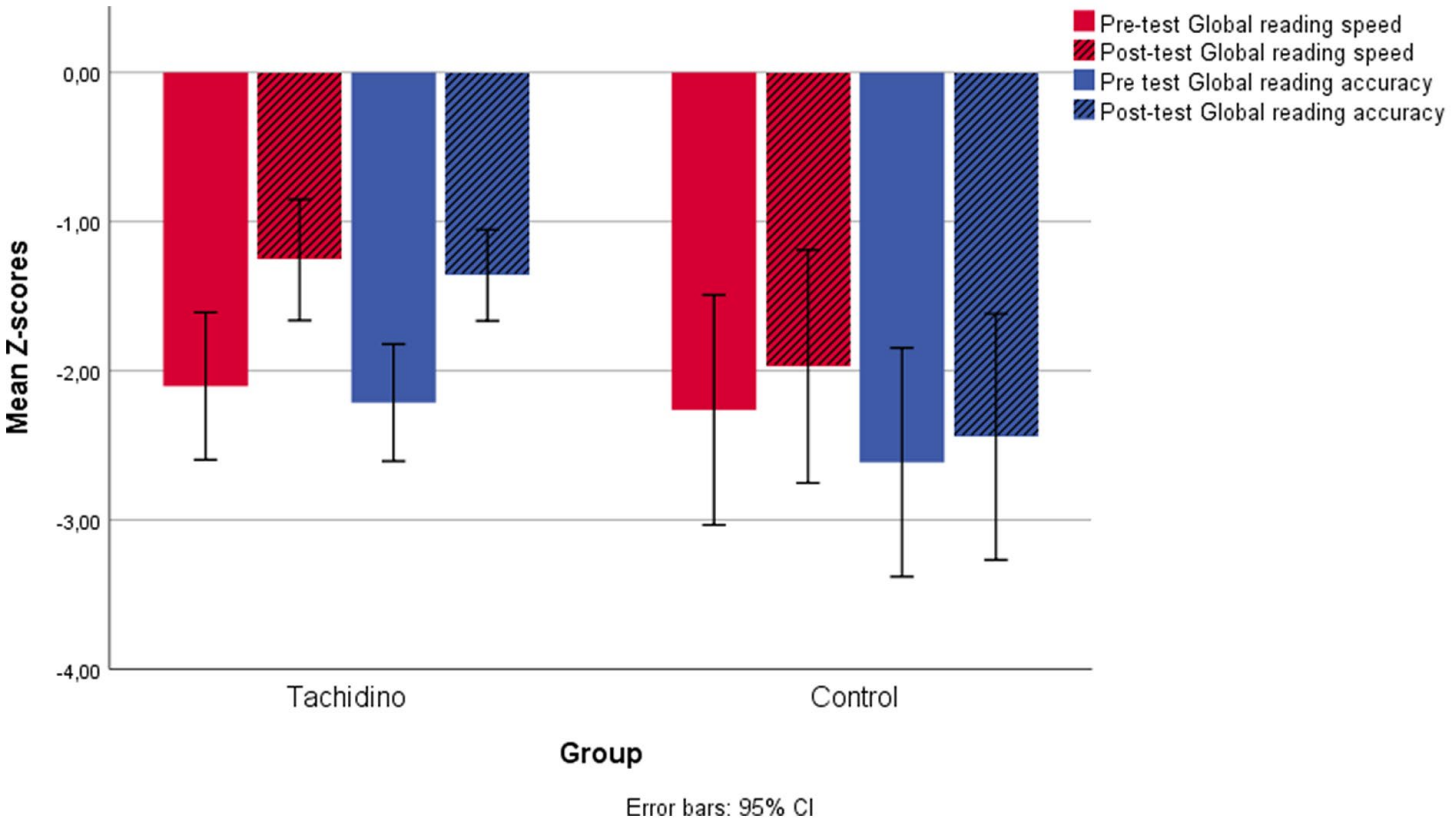
Pre-test and post-test Global reading scores for speed and accuracy in the untreated control group and the Tachidino group.

A series of independent sample *t*-tests were performed to compare age and reading skills of the control group (*n* = 16) to the group of children treated with the Tachidino program, who did not have comorbid ADHD and had not been involved in other clinical intervention programs for learning disorders in the past (*n* = 82). The analyses showed a significant age difference between the two groups (*t* = −3.05, *p* = 0.003). Repeated-measures ANOVAs on pre-and post-treatment scores, with group as a between-subject variable and age as covariate, showed a significant interaction between treatment and group for both Global reading speed and Global reading accuracy [*F* (1, 95) = 4.33, *p* = 0.040, η^2^_p_ = 0.044; *F* (1, 95) = 5.84, *p* = 0.018, *η*^2^_p_ = 0.058]. Post-hoc tests on the two groups separately, comparing pre-and post-treatment scores showed a significant improvement in reading speed for both groups (all *p_s_* < 0.035), while reading accuracy differed only in the group who underwent the Tachidino treatment (*p* < 0.001). Figure 1 shows pre-test and post-test Global scores for speed and accuracy in the two groups (Figure 1). Post-hoc paired-samples *t*-tests comparing pre-with post-treatment scores in the single components of the Global scores (text, word and nonword reading) for each specific parameter (speed or accuracy) showed significant improvement from pre-to post-test in text reading speed in both groups (pre-test *p* < 0 0.001 in the Tachidino group and *p* < 0.003 in the control group), in word and nonword reading speed in the Tachidino group only (all *p_s_* < 0.001), and in text, word and nonword reading accuracy in the Tachidino group only (all *p_s_* < 0.001). Means and standard deviations of pre-and post-test scores are reported in Table 1.

**Table 1:** Mean scores and standard deviations of pre-and post-test for the Tachidino and the control (untreated, waiting list) groups, in the three components of reading scores: text, word and nonword reading.

			Text	Words	Nonwords
			Mean (SD)	Mean (SD)	Mean (SD)
Tachidino group(*n* = 82)	Reading speed	PRE	−1.38 (2.05)	−2,50 (2,21)	−2.14 (2.34)	POST	−0.78 (1.18)	−1.43 (1.93)	−1.36 (2.17)	*t*-test	p < 0.001	*p* < 0.001	*p* < 0.001	Reading accuracy	PRE	−1.41 (1.51)	−2.81 (2.62)	−2.26 (1.71)	POST	−0.80 (1.18)	−1.75 (1.89)	−1.47 (1.72)	*t*-test	p < 0.001	*p* < 0.001	*p* < 0.001
		Control group(*n* = 16)	Reading speed	PRE	−1.70 (0.81)	−2.85 (2.13)	−2.23 (1.79)	POST	−1.48 (0.95)	−2.54 (2.04)	−1.88 (1.71)	*t*-test	*p* = 0.003		n.s.	n.s.	Reading accuracy	PRE	−3.75 (1,51)	−2.72 (1.93)	−1.36 (1.32)	POST	−3.56 (1.91)	−2.10 (2.10)	−1.65 (1.71)	*t*-test	n.s.	n.s.	n.s.

### 3.2 ADHD comorbidity

Independent sample t-test showed absence of age and reading/writing performance differences between children with (*n* = 17) and without (*n* = 104) a diagnosis of ADHD (all *p_s_* > = 0.349). Dyslexia subtype distribution was comparable in the two groups (chi-square = 3.927, *p* = 0.269). When comparing pre-and post-treatment performances with repeated measures ANOVAs with treatment as a within-subject factor and ADHD as a between-subject factor, a significant main effect of treatment on global reading speed, global reading accuracy, and word writing accuracy confirmed treatment effectiveness [*F* (1, 100) = 41.59, *p* < 0.001, *η*^2^_p_ = 0.294; F (1, 100) = 33.26, *p* < 0.001, *η*^2^_p_ = 0.250; *F* (1, 92) = 8.76, *p* = 0.004, *η*^2^_p_ = 0.087, respectively].

For global reading speed and accuracy and word writing accuracy the analyses showed no significant interaction between treatment and ADHD comorbidity (all *p_s_* > 0.569). No ADHD effects emerged for any measure (all *p_s_* > 0.249).

Equivalence testing through TOST independent samples *t*-tests confirmed substantial equivalence of the results of the two treatments for reading speed, whereas the results were less clear-cut for reading accuracy and word writing accuracy. So, the test allowed rejection of the hypothesis that the true effect was smaller than *d* = −0.5 or larger than *d* = 0.5, with a significant result for the test against ΔL/ΔU for reading speed, *t* (24.2) > = 1.73, *p* < = 0.048; for reading accuracy, *t* (18.3) was significant with respect to ΔU (*t* = −2.24, *p* = 0.019) but not to ΔL (*t* = 1.21, *p* = 0.121), while the opposite was true for writing accuracy where *t* (16.0) was significant against ΔL (*t* = 2.19, *p* < 0.022) but not against ΔU (*t* = 1.28, *p* = 0.110).

**Figure 2:**
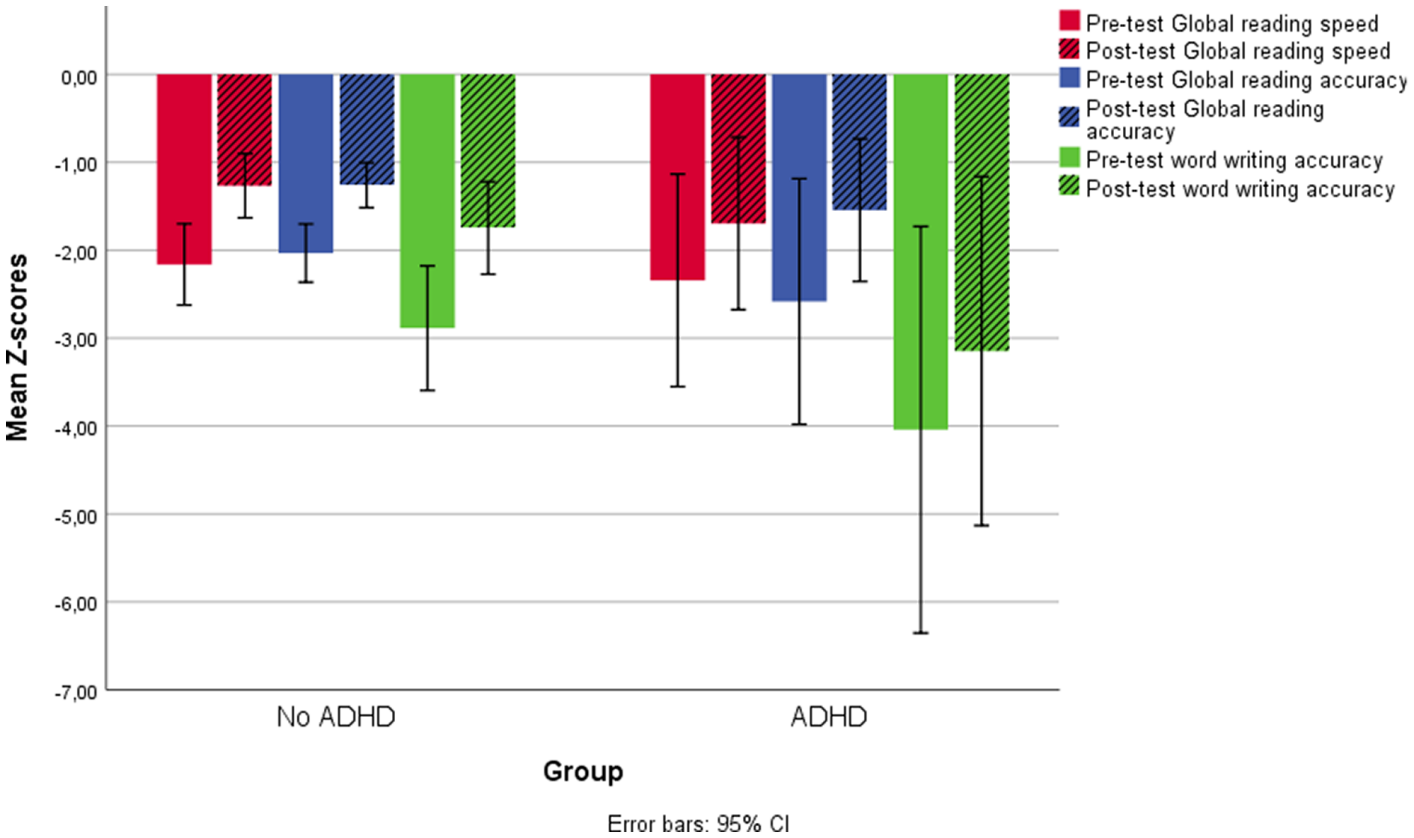
Pre-test and post-test Global reading scores for speed and accuracy and word writing accuracy in the two groups of children with and without ADHD comorbidity.

Figure 2 shows pre-test and post-test Global scores for reading speed and accuracy and for word writing accuracy in the two groups (Figure 2).

### 3.3 Previous treatment with SLT

A preliminary analysis with an independent sample *t*-test showed no age differences between children with (*n* = 22) and without (*n* = 114) previous treatment with SLT (*p* = 0.375). However, the same analysis showed a significant difference between the two groups in word writing scores before the beginning of the Tachidino treatment (*t* = −2.57, *p* = 0.014). Global reading speed and accuracy, instead, did not differ before intervention (all *p_s_* > 0.083). Comparing improvements in reading ability with repeated-measures ANOVAs, due to the predicted difference in effectiveness in favor of the not previously treated group, a unidirectional hypothesis could be applied in this case, with one-tailed *p*-values (alpha = 0.1). No significant differences emerged between the two groups for reading speed [*F* (1, 134) = 0.191, *p* = 0.663], but a significant difference emerged for reading accuracy under a unidirectional hypothesis [*F* (1, 134) = 2.79, *p* = 0.098]. ANOVA analysis with pre-Tachidino word writing scores as covariate showed a significant effect of the covariate with respect to the writing gains [*F* (1, 125) = 79.90, *p* < 0.001, *η*^2^_p_ = 0.390], but no significant differences in improvement were found between the two groups (*p* = 0.344). A series of paired sample t-tests were used to assess differences between pre-and post-treatment with Tachidino in the two groups separately. Analyses showed significant improvements in reading speed and accuracy and writing accuracy, for both groups (all *p_s_* < 0.022). A post-hoc analysis on the different components of Global reading accuracy showed that the difference between the two groups depended on nonword accuracy scores [*F* (1,130) = 6.626, *p* = 0.011].

**Figure 3:**
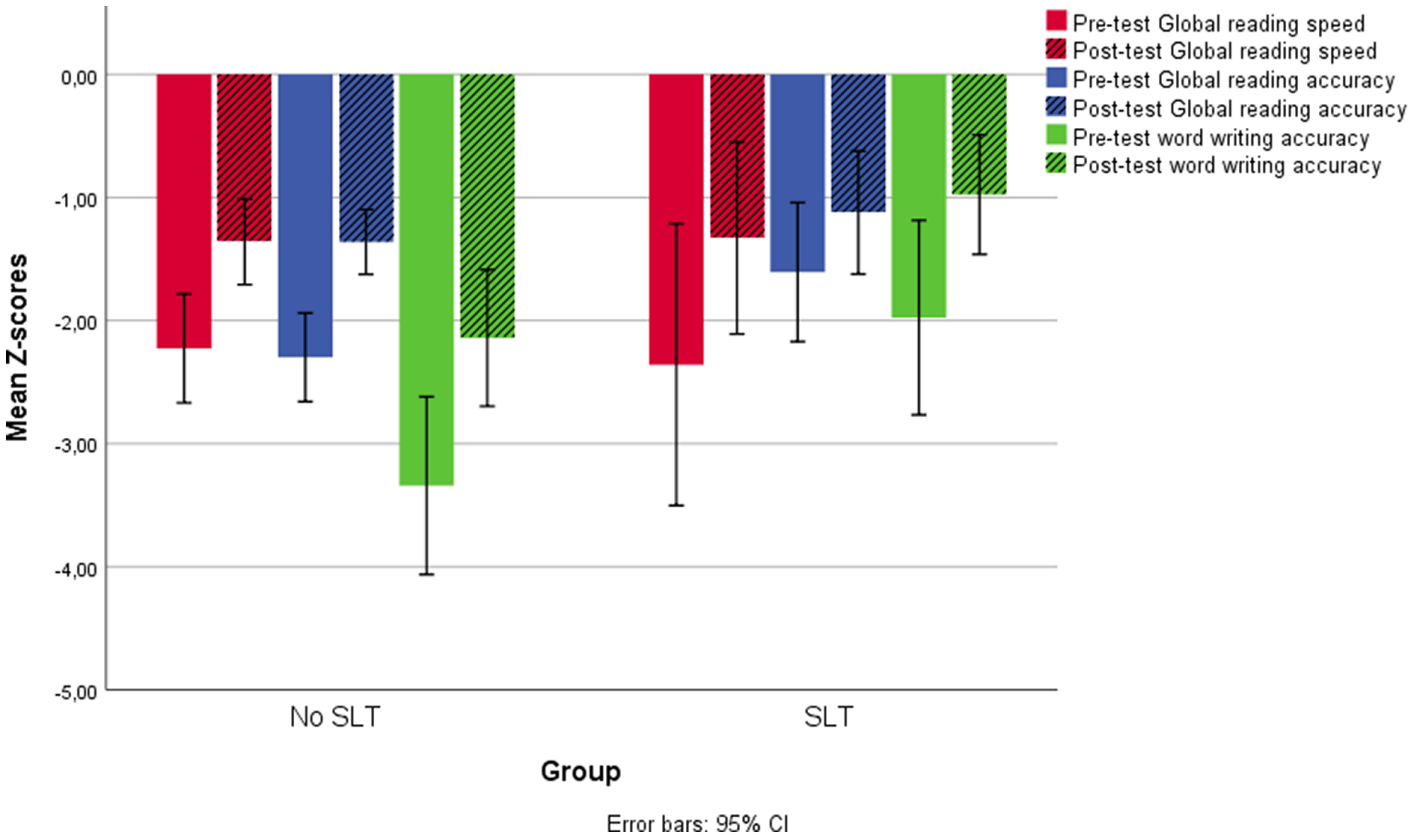
Pre-test and post-test Global reading scores for speed and accuracy and word writing accuracy in the two groups of children with and without a clinical history of SLT.

**Figure 4:**
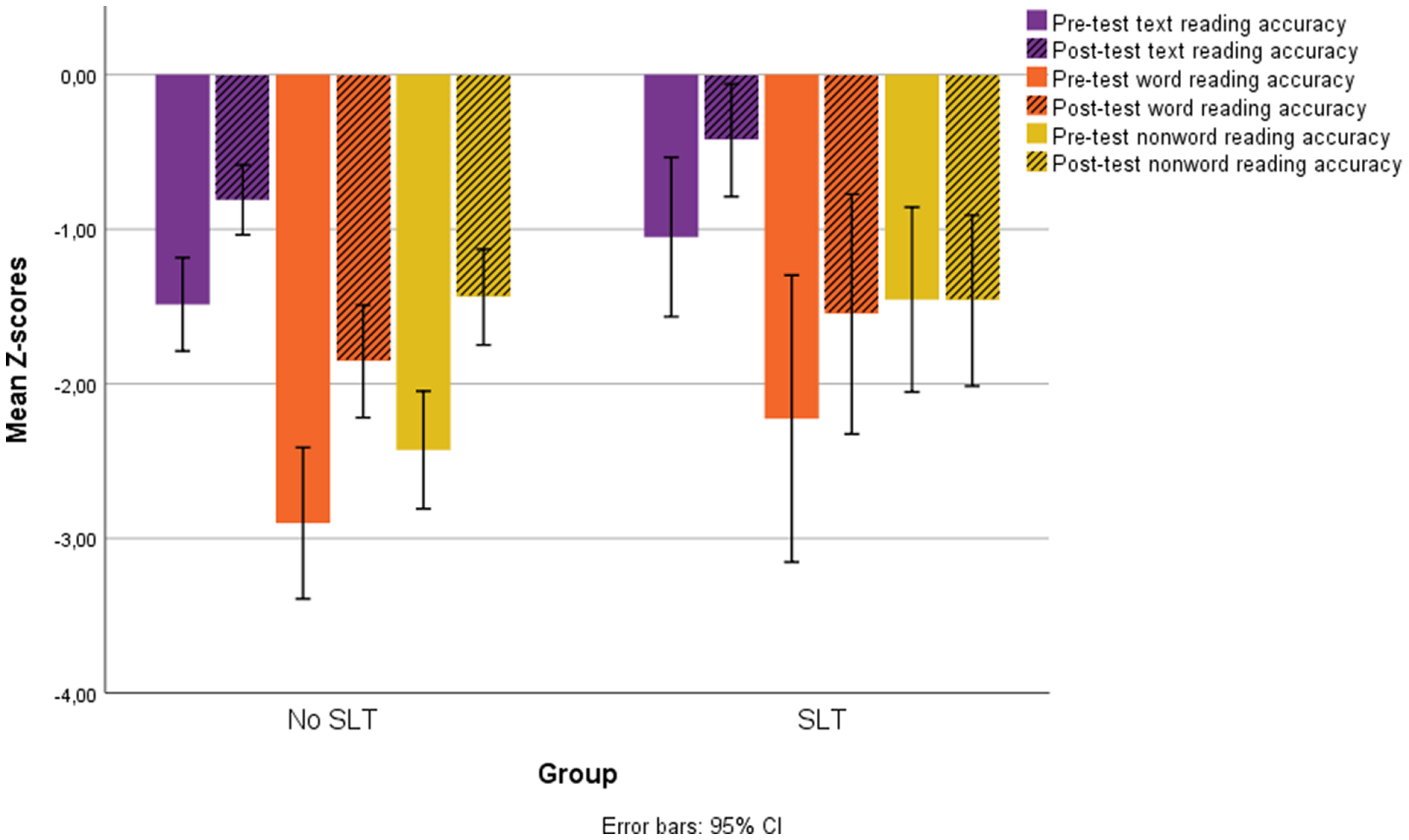
Pre-test and post-test accuracy scores for text, word and nonword reading in the two groups of children with and without a clinical history of SLT.

Figure 3 shows pre-test and post-test Global scores for reading speed and accuracy and for word writing accuracy in the two groups. The pre-and post-test reading accuracy scores for the two groups on all subtests are shown in Figure 4.

### 3.4 Tachidino treatment repetition

Preliminary analyses comparing pre-test scores in the group of children who had (*n* = 30) and who had not (*n* = 84) received repeated treatment (excluding those who had undergone SLT) showed no significant differences for age and baseline levels (all *p_s_* > 0.83).

Furthermore, the comparison of post-test scores for the first cycle in the children who had and had not received repeated treatment showed significant differences in post-test accuracy scores for word reading speed (*t* = 2.10, *p* = 0.038) and nonword reading accuracy (*t* = 2.34, *p* = 0.021).

Finally, the comparison of difference-scores during the first cycle for children who had and who had not received repeated treatment showed significant differences in difference-scores for nonword reading accuracy only (*t* = 2.01, *p* = 0.047).

The mean scores and standard deviations of the two groups for the pre-and post-treatments of the first and second cycles of Tachidino are reported in Table 2.

**Table 2:** Pre-and post-treatment mean scores and standard deviations obtained during the first and second cycle of Tachidino.

		First Tachidino cycle		Second Tachidino cycle
		One cycle (*n* = 84)	Two cycles (*n* = 30)	Two cycles (*n* = 30)
		Mean (SD)	Mean (SD)	Mean (SD)
Global reading speed	PRE	−2.21 (2.44)	−2.45 (1.94)	−2.30 (1.52)
	POST	−1.26 (1.88)	−1.76 (1.57)	−1.27 (1.55)
Global reading accuracy	PRE	−2.23 (1.73)	−2.64 (2.30)	−2.15 (1.78)
	POST	−1.25 (1.40)	−1.82 (1.35)	−1.47 (1.53)
Word writing accuracy	PRE	−3.05 (3.60)	−4.13 (4.14)	−3.96 (5.48)
	POST	−1.89 (2.46)	−2.82 (3.80)	−3.02 (4.25)

Data are presented separately for the two groups of children who have and who have not received repeated treatment.

A series of paired-samples t-tests conducted for the group of children who underwent two treatment cycles with the Tachidino program showed significant reading and writing improvements after the first cycle (all *p*s < 0.003). Moreover, significant reading improvements were shown also after the second cycle of treatment (all *p*s < 0.001). Improvement in writing accuracy, instead, was not significant after the second cycle of Tachidino (*t* = −1.59, *p* = 0.124).

**Figure 5:**
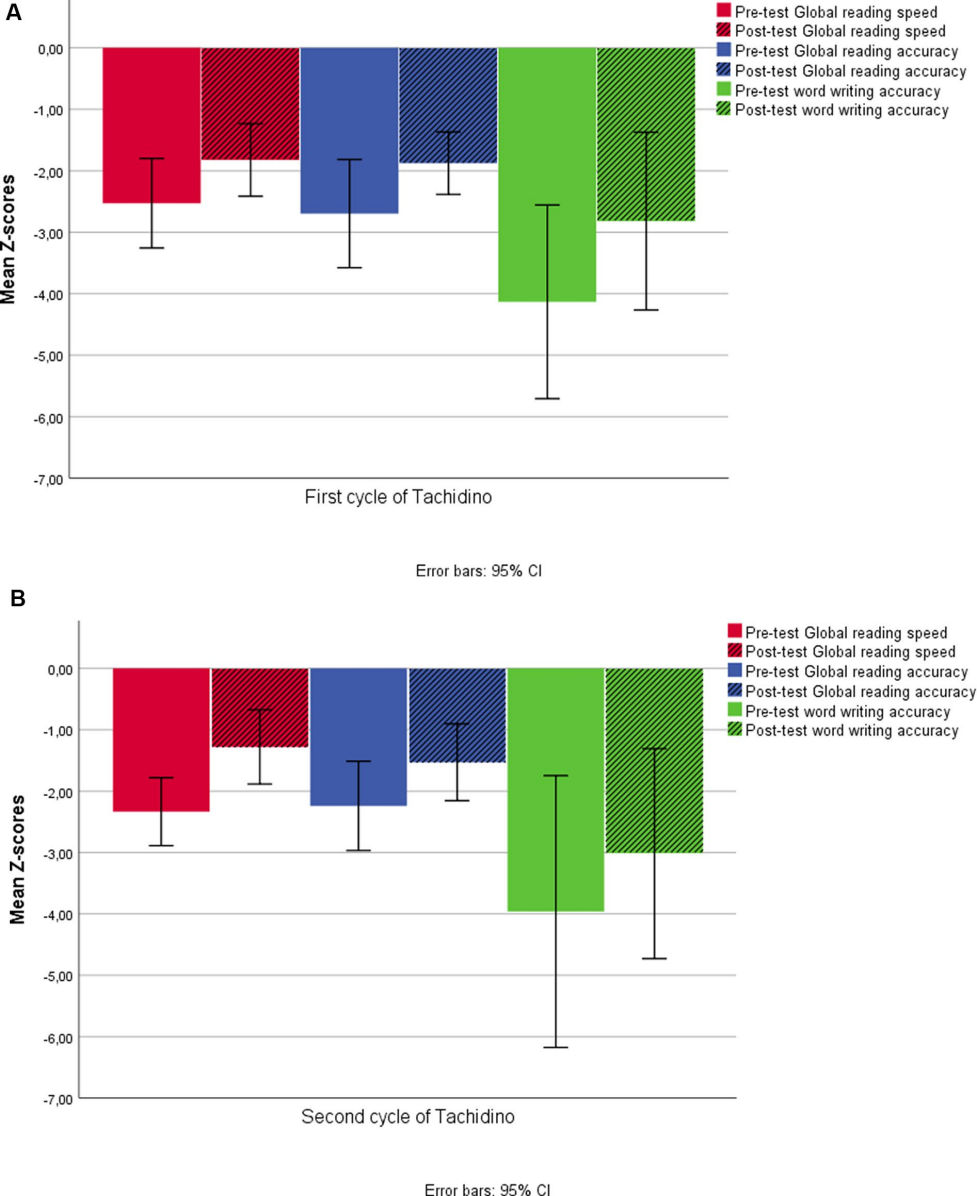
Pre-test and post-test Global reading scores for speed and accuracy and word writing accuracy after the first cycle of Tachidino **(A)** and after the second cycle of the treatment **(B)**. Data are referred only to the group of children who repeated the treatment.

Reading and writing improvements after the first cycle of Tachidino were compared to those obtained after the second cycle of the treatment program. Specifically, the three Difference scores (between the post-and the pre-treatment scores) of the first cycle were compared to the three Difference scores obtained in the second cycle of Tachidino treatment. Analyses were computed only in the group of children which received Tachidino treatment twice. A repeated-measures ANOVA did not show any significant main effect of treatment repetition for global reading speed, global reading accuracy, or word writing accuracy (all *p_s_* > 0.142). Figure 4 shows pre-test and post-test Global scores for reading speed and accuracy and for word writing accuracy in the 2 cycles of Tachidino treatment (Figures 5A,B).

## 4 Discussion

The aim of the present study was to investigate the effects of possible sources of variation in the effectiveness of remote treatment of dyslexia with the Tachidino platform. More specifically, we compared the gains in reading and in writing skills between different groups of children with DD belonging to different clinical conditions. In particular, we focused on possible effects of ADHD comorbidity on the outcomes of the Tachidino treatment, as well as on the impact that previous treatments (such as SLT or repeated cycles of the Tachidino program) could have on its effectiveness.

All children who participated in the study, except for the control group who was on a waiting list, had received a 3–4 weeks - treatment with Tachidino, a web-based program for neuropsychological intervention in developmental dyslexia (2). Pre-post changes in reading and writing abilities were assessed and compared between different groups of children. As expected, all participants significantly improved in reading and writing after treatment with the Tachidino program, and these improvements were significantly larger than those observed in the untreated control group, who showed significant improvement from pre-test to post-test in text reading speed only, presumably due to familiarization with the text to be read. This result confirms what was found in previous studies on the effectiveness of the program, additionally showing that improvement cannot be simply attributed to test–retest effects even if assessment is repeated at a short time distance (treatment duration is between three and 4 weeks).

Moreover, irrespective of comorbidity, clinical history and treatment repetition, significant benefits from the Tachidino treatment have been confirmed. The Tachidino treatment appeared to be effective in improving reading speed and accuracy and writing accuracy in children with or without ADHD comorbidity, in children with a clinical history of speech and language therapy, and in children who repeated the treatment. The gains in learning abilities in the ADHD group were not different from those of the group of children without comorbidity, especially for what concerns reading speed. As to reading accuracy, it is not possible to exclude that the treatment is even more effective for children with ADHD; in the case of writing accuracy, instead, even if the results are still comparable (not statistically different) it is not possible to claim that effectiveness is completely equivalent in the two groups. This result allows us to confirm the results of previous studies employing different methodologies for reading intervention (25, 28, 29). The nature of the attentional component of the Tachidino treatment, addressing both decoding and visual–spatial attention, appears to be effective for the stimulation of attentional functions specifically impaired in children with a diagnosis of ADHD. Moreover, the remote feature of the mode of delivery of the treatment, requiring the child to organize and control independently their own activity seems not to have any detrimental effect on these children, and to be perfectly manageable. A rigorous and intensive decoding treatment such as the Tachidino program can thus be considered an eligible method for intervention in children with ADHD and for at-risk readers with ADHD (20).

As to the comparison with the group of children with a clinical history of SLT, improvements after treatment with Tachidino did not differ between the group of children with or without a history of SLT, except for reading accuracy, more specifically for nonword reading. Indeed, at an *ad-hoc* check it turned out that the two groups differed in nonword reading accuracy already at pre-treatment assessment [*t* (130) = −2.117, *p* = 0.036], with mean *z*-score for the SLT group = −1.45 versus −2.43 in the Tachidino group, so that it can be argued that reading accuracy had already been stimulated during SLT treatment and there was less space for further improvement. Since SLT addressing phonological awareness may be expected to produce improvement in reading accuracy especially stimulating the indirect route of reading through grapheme-to-phoneme conversion, a post-hoc analysis on data retrieved from clinical records was performed to check for baseline differences in phonological performance. Children of the SLT group in fact showed (at the very first assessment prior to SLT) a significantly higher number of errors (mean errors = 6.42) in phonological awareness tests - phoneme blending and phoneme elision (47) - than the group of children without a history of speech and language therapy (mean errors at pre-test before Tachidino treatment = 4.01, *t* = 3.55, *p* = 0.001). The severe impairment in phonological decoding abilities could explain why these children were not immediately offered the Tachidino treatment. Unfortunately, the absence of data at pre-test and post-test assessment for this group does not allow us to make any inference as to the relationship between phonological awareness scores and reading accuracy before starting the treatment with Tachidino, nor to the improvement of phonological awareness through the SLT intervention. Overall, though, it can be concluded that the choice of offering SLT intervention before Tachidino allowed these children to reach a similar level of reading and writing performance at the end of the Tachidino treatment as the group who had not received any preliminary treatment.

Contrary to expectations, the effectiveness of Tachidino treatment was not significantly reduced in the second versus the first cycle of intervention, even if word writing accuracy after the second cycle of Tachidino did not show any significant improvement. More precisely, it can be observed that, as expected, the children who underwent a second cycle with the Tachidino program did not differ from the other children in pre-test scores (before the first cycle) nor in the degree of improvement during the first cycle. This means that repetition of treatment should not be considered as a means to reach clinical goals in children who seem to respond less to the treatment (the issue of non-responders should be addressed in *ad-hoc* studies), but rather as a means to gain further benefits in children who already show good (or average) response to treatment. In other terms, one can conclude that the treatment does not loose efficacy (neither in a cognitive-neuropsychological nor in a motivational perspective) after the first application. Moreover, the children who underwent a second cycle of treatment showed scores at post-test (after the first cycle) in word writing accuracy still below the level of clinical attention. For this reason, it is reasonable to presume that they were offered a second treatment focusing on writing more than on reading skills. Indeed, at the end of the second cycle, their writing scores had changed from almost 4 to about 3 standard deviations below age mean (i.e., an average improvement of about 1 z-score during the second cycle), even if this improvement was not statistically significant due to high variability. This suggests that, even if the treatment with the Tachidino program was found to be effective on writing skills in the large group, children who have a very severe impairment in spelling may need to work longer or with additional strategies in order to reach an acceptable level of writing performance. Nonetheless, it should be noticed that their reading scores had further improved (all were above-1.5 standard deviations with respect to age norms) during the second cycle of treatment.

Limitations of the present study include sporadic missing data, due to technical or recording problems or, in the case of writing/spelling tests, to changes in clinical protocols in different periods (due to the necessity of reducing assessment time during the COVID-19 pandemic). Nonetheless, the very positive results of the study allow clear conclusions to be drawn on the effectiveness of remote treatment for DD, independent of clinical variables such as the presence of comorbidity with ADHD and the repetition of treatment. This encourages use of remote techniques that may be very advantageous both in terms of treatment efficiency (reducing the costs and efforts related to the delivery of treatment without reducing the efficacy of treatment itself) and in terms of risk reduction in a period where pandemic-related dangers call for special caution in accessing public health structures. The special advantages of web-based intervention will probably be more evident in the future, when the large amounts of data systematically collected by research centers may help shed further light on the mechanisms of improvement and their relations with initial clinical profiles, in the perspective of a more and more personalized approach to intervention also for neurodevelopmental disorders. Future improvements may further employ more innovative technologies such as the use of VR in the treatment, with the aim of making user experience more and more rewarding and to keep the child’s engagement as high as possible (but carefully selecting low-cost, non-demanding technology to avoid a reduction in accessibility and sustainability of treatment for the users). The gradual introduction of more sophisticated technologies in the future will also provide further information about the impact that motivation and engagement through gamification (48, 49) have on treatment effectiveness.

## Data availability statement

The dataset of the study has been deposited in the Zenodo repository (DOI: 10.5281/zenodo.7497190) and will be made available upon written request to the first author (public sharing is not allowed by the Ethical Committee).

## Ethics statement

The studies involving humans were approved by the Ethics Committee of the Scientific Institute IRCCS *E. Medea* (Comitato Etico dell’IRCCS Eugenio Medea – sez. Scientifica dell’Associazione “La Nostra Famiglia”). The studies were conducted in accordance with the local legislation and institutional requirements. Written informed consent for participation in this study was provided by the participants’ legal guardians/next of kin.

## Author contributions

MLL: conceptualization, methodology, validation, formal analysis, data curation, writing-original draft, visualization, and supervision. FB: formal analysis, data curation, visualization, and writing-original draft. PM and MGL: investigation, data curation. AS: resources and validation. ST: methodology and data curation. MM: conceptualization, resources, project administration and funding acquisition.
